# Visual screening in an orthogeriatric rehabilitation setting: a feasibility evaluation

**DOI:** 10.1093/geroni/igag022

**Published:** 2026-03-16

**Authors:** Martin Chi Kit Yan, Siraj Farid, Jay Chillala, Robert A Harper

**Affiliations:** Department of Geriatric Medicine, Trafford General Hospital, Manchester, United Kingdom; Department of Optometry, Manchester Royal Eye Hospital, Manchester, United Kingdom; Department of Geriatric Medicine, Trafford General Hospital, Manchester, United Kingdom; Department of Geriatric Medicine, Trafford General Hospital, Manchester, United Kingdom; Department of Optometry, Manchester Royal Eye Hospital, Manchester, United Kingdom; Faculty of Biology, Medicine and Health, University of Manchester, Manchester, United Kingdom

**Keywords:** Vision health, Patient safety, Preventive care, Rehabilitation care, Socioeconomic status

## Abstract

**Background and Objectives:**

Vision loss is a significant risk factor for falls. Given the associated morbidity and mortality, proactive risk-reduction strategies are warranted. This evaluation aimed to assess the feasibility and potential clinical value of bedside visual screening in an orthogeriatric rehabilitation setting and to estimate the proportion of previously unrecognized visual deficits among older inpatients admitted following a fall.

**Research Design and Methods:**

In this prospective, single-center feasibility evaluation, 21 patients aged ≥65 years admitted post-falls were assessed. Eligible participants scored >6 on the Abbreviated Mental Test Score. Bedside assessments included unaided vision, visual acuity (VA), low-contrast VA, and visual fields using Logarithm of the Minimum Angle of Resolution (LogMAR) charts and the Melbourne Rapid Fields web-based platform. Ocular history and participant feedback on the screening experience were also collected.

**Results:**

Unaided vision or habitual VA worse than 0.3 LogMAR (<6/12 Snellen equivalent) in at least one eye was observed in 67% of participants; 88% showed impaired low-contrast VA. Only 33% had been examined by a primary care optometrist in the year preceding admission. A proportion of deficits were potentially modifiable. Screening was well tolerated, with participants reporting a good understanding of the procedures.

**Discussion and Implications:**

Bedside visual screening by trained non-ophthalmic specialists is feasible and potentially clinically informative in the orthogeriatric setting. The high proportion of undetected visual deficits suggests missed opportunities for falls prevention. Routine standardized visual assessments may enable timely referrals, improve visual outcomes, and reduce falls-related morbidity in older adults.

Innovation and Translational Significance: This is the first evaluation known to the authors to implement a hybrid visual screening model utilizing a LogMAR chart and web-based perimetry in an orthogeriatric rehabilitation setting. Bedside screening conducted by non-ophthalmic clinicians proved feasible and revealed a high prevalence of previously unrecognized visual deficits, many of which were potentially modifiable. Embedding such a model into routine inpatient care could strengthen falls prevention strategies, support timely referral to community optometry or ophthalmology services, and inform development of integrated screening pathways to improve outcomes in older adults.

## Background and objectives

Falls are the second leading cause of accidental death worldwide, primarily affecting older adults aged 65 years and above (Mehta et al., 2022). They are also a major contributor to morbidity and mortality in this population, frequently leading to fractures, prolonged hospitalization, deconditioning, and increased risk of institutionalization.

In the United Kingdom (UK), approximately one in five individuals over the age of 75 experiences some degree of vision loss (Evans et al., 2004). The prevalence of age-related ocular conditions, such as cataract, macular degeneration, and glaucoma, is expected to increase with age, presenting increasing challenges for health systems focused on geriatric care. Among these conditions, visual field loss, particularly from glaucoma, has been strongly linked with an increased risk of falls and related injuries (Black et al., 2011; Freeman et al., 2007; Ramulu et al., 2012; Tsang et al., 2024). Reduced vision may impair hazard detection, compromise spatial navigation, and hinder balance, with this problem being more evident in unfamiliar or cluttered environments. Despite this relationship between reduced vision and falls, many patients with impaired vision remain undiagnosed in the community. As a result, older individuals who are at high risk of falling may continue living with associated and potentially contributory visual deficits without appropriate interventions (Cedrone et al., 2008; Varma et al., 2011).

Poor vision has been demonstrated to contribute to up to 30% of falls in the UK (College of Optometrists and British Geriatric Society, 2020). Studies conducted by Jack et al. (1995) and Cox et al. (2005) showed that up to 75% of visual impairment-related falls admissions are correctable, either with suitable spectacle correction or cataract surgery. Despite this association, routine vision screening is not typically embedded within acute or rehabilitation care pathways for older patients, particularly outside of specialist ophthalmology services. For example, in Greater Manchester, where this evaluation was conducted, there is currently no formal visual screening program for older adults following a fall, either in inpatient settings or in community rehabilitation services. Current provision relies on patients attending community optometrists on their own initiative, often at annual or biannual visits. However, mobility issues, cognitive impairment, and lack of awareness of benefits and entitlements may limit uptake, even among those eligible for NHS-funded sight tests under the General Ophthalmic Service (GOS).

Many non-ophthalmic specialists in the UK report being uncomfortable or apprehensive about conducting formal vision assessments (Mendall et al., 2024). A simplified bedside visual screening tool may support clinicians by enabling early identification of high-risk patients and prompting appropriate referral to eye care services for assessment and intervention. Visual screening tools, including visual acuity charts and computer-based perimetry, may be particularly useful in this context.

This pilot evaluation aimed to evaluate the feasibility of implementing structured bedside visual screening in an orthogeriatric rehabilitation setting. It also aimed to estimate the proportion of previously unrecognized visual deficits among older inpatients following a fall, and to explore how simple, standardized assessments, administered by non-ophthalmic clinicians, could contribute to falls prevention strategies through timely referral to specialist care. In achieving these aims, this evaluation may support the development of an integrated visual screening pathway in collaboration with primary care optometrists, with potential implications for future policy and quality improvement initiatives across the region and beyond.

## Research design and methods

### Evaluation aim, design, and setting

This prospective, single-center feasibility evaluation was conducted at the Early Limb Mobilisation Unit at Trafford General Hospital, part of Manchester University NHS Foundation Trust. The primary aim was to evaluate the feasibility of bedside visual screening in older adults admitted following a fall, and to explore the extent of previously unrecognized visual impairment within this population.

### Characteristics of participants

Participants were included if they were aged 65 years or older, admitted following a documented fall, with an Abbreviated Mental Test Score (AMTS) greater than 6 out of 10 (Hodkinson, 1972), and who were able to provide informed verbal consent indicating willingness to participate in the feasibility project. Exclusion criteria included severe cognitive impairment (AMTS ≤6), acute delirium, inability to engage with visual testing (e.g., due to aphasia, being bedridden, or extreme fatigue), and/or being clinically unwell (e.g., due to infection). Recruitment took place between January 2025 and April 2025.

### Consent and ethical considerations

Verbal informed consent was obtained from all participants prior to their involvement in the evaluation. This feasibility project was conducted as a quality improvement initiative within routine clinical care, and as such, formal ethical approval was not required according to local governance procedures. Ethical decision tools from both the NHS Health Research Authority (2024) and the University of Manchester (2024) were completed and confirmed that formal approval was not necessary.

### Screening processes

Visual assessments were carried out at the bedside by a resident doctor who had received individual competency training from an experienced ophthalmic clinician at Manchester Royal Eye Hospital. The training comprised of three components: (1) written training pack outlining the screening protocol, visual assessment principles, and interpretation of common findings, accompanied by a self-assessment exercise; (2) two short face-to-face practical training sessions covering examination techniques, use of the screening tools, recognition of reliable test performance, and the appropriate endpoint for visual threshold; and (3) observed practice with feedback to support competency prior to independent assessments. The total duration of training was approximately three hours.

Standardized screening tools and protocols were used throughout. Visual acuity (VA), a measure of visual resolution, was assessed using an internally illuminated ETDRS LogMAR chart at a distance of 3 m under general photopic ward lighting conditions. Unaided vision, habitual (i.e., usual spectacles) VA and pinhole-corrected VA, where applicable, were measured for each eye. Visual fields (VF) assess the ability to detect visual stimuli in the peripheral field of vision. Low-contrast visual acuity (LCVA) reflects visual performance under conditions of reduced contrast and low luminance. VF and LCVA were evaluated using Melbourne Rapid Fields (MRF), a validated web-based tool for perimetry and contrast sensitivity testing, which has demonstrated agreement with the Humphrey Field Analyser for VF testing (Prea et al., 2018).

The global index of VF status, Mean Deviation (MD), was used as a measure of VF loss. Test reliability indices of VF status, including fixation loss (failure to maintain gaze on the central target), false positive responses (responses made when no stimulus is presented) and false negative responses (an indicator of inattention, defined as failure to respond to stimuli previously detected), were used to assess the validity of VF results, in line with standard perimetric practice (Heijl, 2021).

Ocular history was obtained during the screening process, including information on spectacle use and adherence, the date of the most recent community optometry visit, and any known ophthalmic diagnoses or follow-up, where applicable. In addition, electronic health records were reviewed to identify any ophthalmic assessments documented since admission. Participants’ postcodes were analyzed using the English Indices of Multiple Deprivation (IMD), a nationally derived area-based measure of socioeconomic deprivation, to estimate participants’ socioeconomic status and explore potential associations between deprivation decile and prior engagement with primary care optometry services.

To evaluate the feasibility and acceptability of the testing process, a brief post-screening questionnaire was administered, assessing patients’ understanding of the administered tests, perceived level of difficulty, and self-rated performance, providing insight into their perceptions and level of engagement.

A standardized screening proforma was used to facilitate consistent data collection across participants, as shown in Supplementary Material.

### Data analysis

Descriptive statistics were used to summarize participants’ demographic characteristics, VA, LCVA, perimetry findings, and patient-reported data from the ocular history assessment. Unaided vision and VA were analyzed using LogMAR scores, with a threshold of >0.3 (i.e., worse than the Snellen equivalent of 6/12) being used to define visual impairment. VF and LCVA results were categorized according to quantitative outputs from the VF software’s platform. Postcode data were cross-referenced with publicly available IMD data to assign participants’ main home addresses to the corresponding English national deprivation deciles. Reported attendance at community optometry services was also analyzed to explore general patterns of eye care access within this cohort. Statistical analysis was conducted using Microsoft Excel spreadsheets (Microsoft Corp., Redmond, Washington).

## Results

### Participant characteristics and overview

A total of 21 participants were included in the evaluation, with a mean age of 84.0 ± 8.2 years, and a near-equal gender distribution of 9 men (42.9%) and 12 women (57.1%). The cohort was mainly composed of older adults living with frailty and multimorbidity, admitted after a fall. All participants scored greater than 6 on the Abbreviated Mental Test Score. As shown in Table 1, unaided vision or VA worse than 0.3 LogMAR in either eye was observed in 67% of participants. Just under 20% of all participants had unaided vision or VA of 0.3 LogMAR in both eyes. Among those not wearing spectacles, 10% had bilateral monocular visions >0.3 LogMAR, compared to the 27.3% who had bilateral monocular VA worse than >0.3 LogMAR among spectacle wearers. Pinhole testing was conducted when the unaided vision or best-corrected VA was >0.2 LogMAR (i.e., worse than 6/9). Of the 16 patients who had pinhole assessment performed, 37.5% showed an improvement of 0.2 LogMAR or greater. LCVA was assessed under low luminance conditions. In this cohort, 88.2% (*n *= 17) demonstrated impaired LCVA of 6/12 or worse in at least one eye, arguably warranting further investigation in individuals without a known history (Forte et al., 2026; Schneck et al., 2004).

**Table 1:** Summary of demographics and key findings.

Variables	Sample size	Mean ± SD (range)	*n* (%)
**Age (years)**	21	84.0 ± 8.2 (67, 97)	
**Gender**	21		
Male			9 (42.9%)
Female			12 (57.1%)
**Unaided vision or VA of LogMAR >0.3 in either eye**	21		14 (66.7%)
**Unaided vision or VA of LogMAR >0.3 in both eyes**	21		4 (19.0%)
Unaided vision of LogMAR >0.3 in both eyes (non-spectacle wearers)	10		1 (10.0%)
Visual acuities of LogMAR >0.3 in both eyes (spectacle wearers)	11		3 (27.3%)
**Improvement (LogMAR >0.2) of pinhole assessment in either eye (pinhole vs unaided or pinhole vs BCVA)^a^**	16		6 (37.5%)
**Mean deviation**	21	−14.26 ± 9.98 (−33.35, −0.82)	
**Reliability of VF^b^ (when FL indices are available)**	20		17 (85.0%)
**Low-contrast VA worse than 6/12 in at least one eye**	17		15 (88.2%)
**Low-contrast VA worse than 6/12 in both eyes**	17		13 (76.5%)
**Documented ophthalmic screening since admission to orthogeriatric unit**	21		3 (14.2%)
**% of community optometry check in the 12 months prior to admission**	21		7 (33.3%)
**% of community optometry check in the 24 months prior to admission**	21		8 (38.1%)

*Note.* BCVA = best-corrected visual acuity; FL = fixation loss; LogMAR = logarithm of the minimum angle of resolution; VA = visual acuity; VF = visual field.

a Pinhole assessment was only conducted if unaided vision (no regular specs) or best-corrected VA is ≥0.2 (i.e., worse than 6/9).

b Reliable if FL ≤33%.

### VF findings

**Figure 1:**
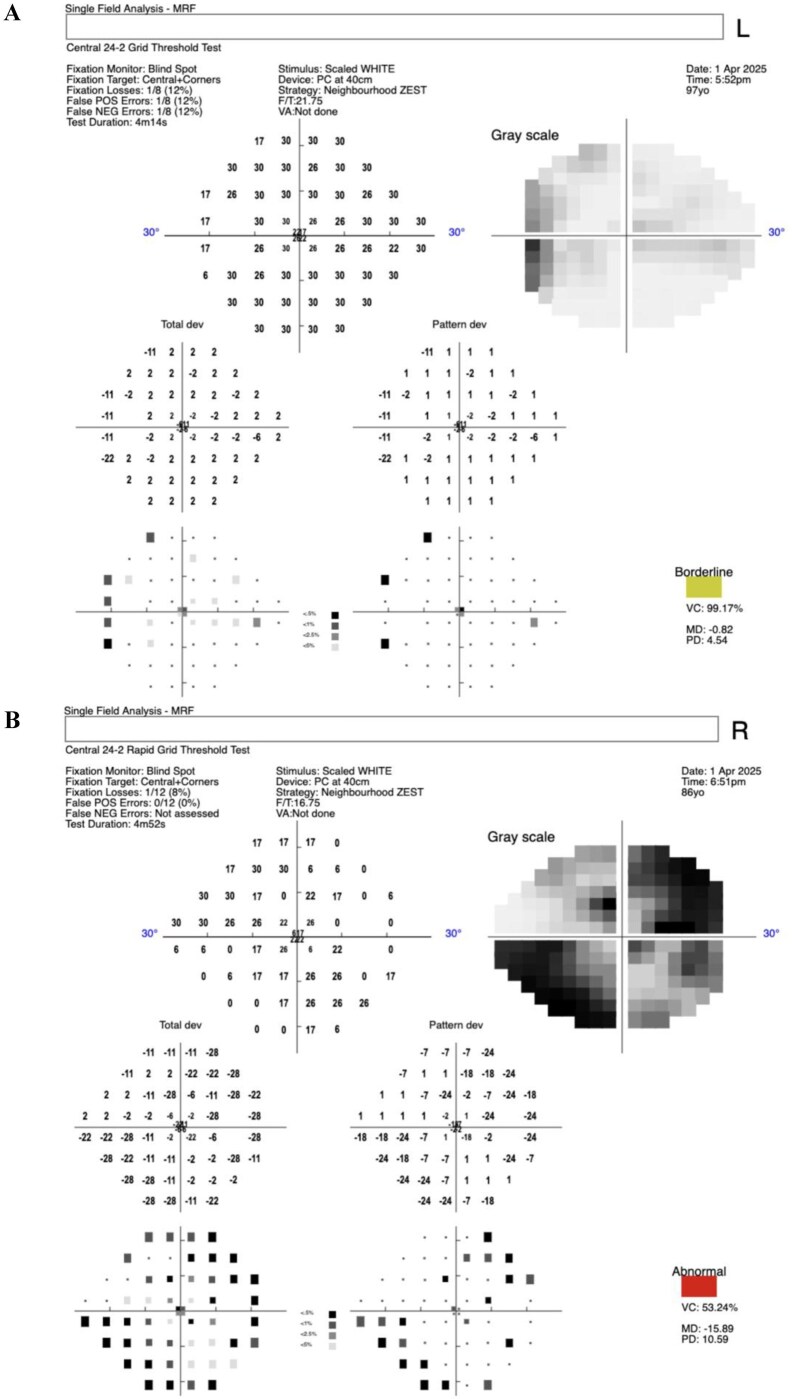
An illustration of VF assessment using MRF, a validated VF screening tool (Glance Optical, 2015). (A) VF report showing no known field abnormalities. (B) VF report showing likely glaucomatous field abnormality. *Note*. VF = visual field; MRF = Melbourne Rapid Fields.

In the 20 patients who had VF assessment performed, 38 eyes underwent perimetry testing. An example of the perimetry testing output in an individual with no known ophthalmic condition compared to an individual with a history of glaucoma and likely defect is shown in Figure 1. Table 1 summarized the mean deviations and reliability outcomes for perimetry results with reliability indices. VF results with Fixation Losses >20%, False Positives or False Negatives >33% were deemed unreliable, with a repeated test being required for those studies (Heijl, 2021; Kumar & Thulasidas, 2020).

### Community optometry engagement and socioeconomic profile

**Figure 2:**
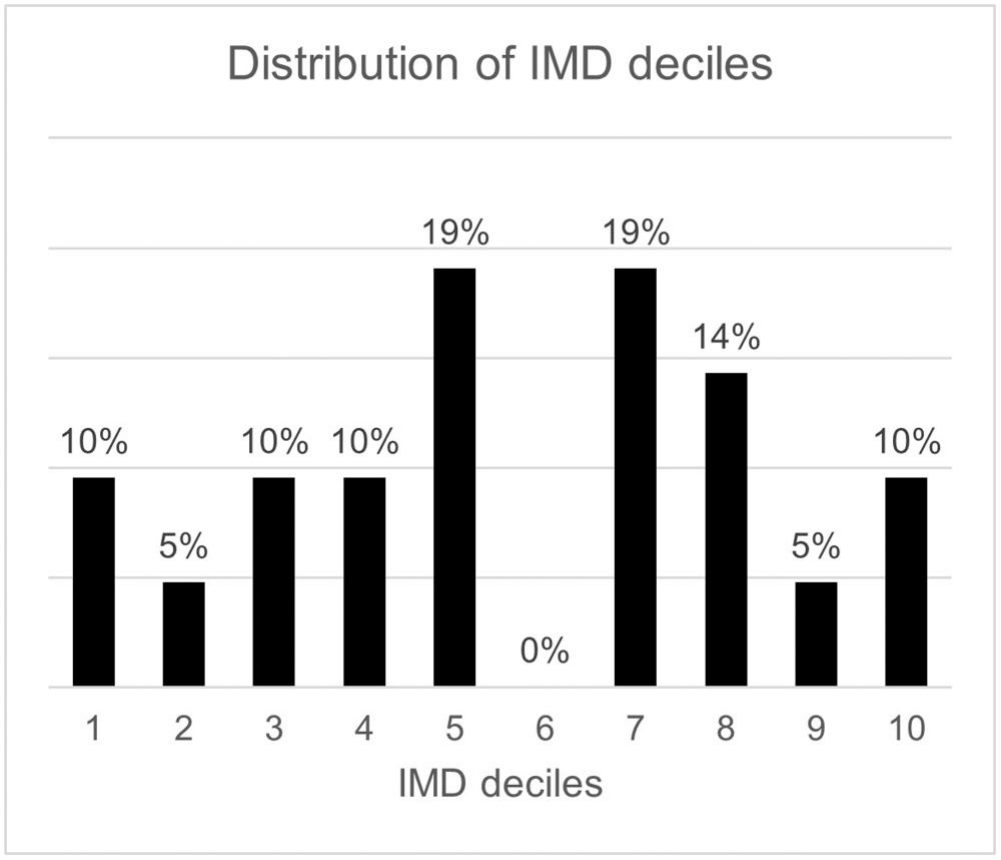
Distribution of IMD deciles, showing the socioeconomic profile of this cohort. *Note*. IMD = Index of Multiple Deprivation.

Reported engagement with community optometry services was limited, with only 33.3% of participants attending within the 12 months prior to admission. The distribution of IMD deciles in this cohort is summarized in Figure 2, with these data revealing that 75% of the cohort resided in the less deprived IMD deciles (4–10). Only 38.1% of participants had had routine sight testing in the previous 24 months, with only 33.3% having had sight testing in the 12 months prior to admission. Interestingly, only one patient in the least deprived three deciles (IMD 8–10) had attended their primary care optometrist for their routine annual eye examination, despite these being freely available under the NHS GOS in England to those over 60 years of age.

### Patient experience

Patients were asked to rate their understanding and perception of the tests using a 5-point Likert scale, where 1 indicated “very poor” and 5 indicated “very good.” Overall, responses were positive, with the mean understanding scores of 4.0 for the VA testing and 3.3 for the VF test. Perceived performance scores were 3.7 for the VA testing and 3.3 for the VF test.

## Discussion and implications

To our knowledge, this is the first evaluation to explore the potential use of structured bedside visual screening in an orthogeriatric rehabilitation setting, with screening being carried out by a non-ophthalmic physician using simple tools (i.e., LogMAR VA chart and a laptop). The evaluation shows that such bedside screening can be feasible, being able to be performed by non-ophthalmic specialists in secondary and intermediate care settings with simple training. The findings revealed a high proportion of previously unrecognized visual deficits in falls-prone older adults, supporting the likelihood of visual impairment being a potentially modifiable falls risk factor.

Reduced VA and LCVA were prevalent even among those without known ocular conditions. These deficits likely reflect uncorrected refractive error and/or potential underlying pathology such as cataract or age-related macular degeneration, and have clear implications for patients’ falls risk, particularly under low luminance conditions or in the presence of uneven surfaces (Evans et al., 2004; Freeman et al., 2007; Sunnen et al., 2008; Tsang et al., 2024). These visual deficits in low-contrast were observed even in some individuals with relatively well-preserved standard VA, reinforcing the potential clinical value of contrast-based screening in falls-prone older adults.

Bedside VF testing appears practical and largely reliable in this cohort, with 85% of tests deemed reliable. While reference-standard validation of visual screening outcomes was not possible in this feasibility cohort, VF results aligned with patients’ clinical histories where known, suggesting utility as a screening tool. For example, Figure 1A demonstrated a near-normal VF, consistent with the clinical background of pseudophakia (previous cataract surgery with intraocular lens implantation) and no other known ocular pathology. Conversely, Figure 1B showed a test outcome exhibiting VF loss, consistent with the patient’s known clinical diagnosis of glaucoma with confirmed retinal nerve fiber layer loss and VF loss on the Humphrey Visual Field Analyser. These findings support the potential utility of web-based VF as a screening tool for peripheral vision loss in older inpatients, reinforcing its potential for use by non-ophthalmic clinicians within ward-based settings. Nonetheless, cognitive demands and patient fatigue during testing remain challenging in some patients.

Strikingly, despite most participants residing in less deprived areas, only one-third had accessed community optometry in the previous year, with less than 40% being tested in the previous 24 months. This finding suggests that barriers to primary eye care may go beyond socioeconomic factors alone, and may include, for example, reduced mobility, impaired cognition, and limited awareness of NHS GOS entitlement. These findings also challenge the assumptions that affluence equates to optimal healthcare engagement, arguably highlighting the need for more proactive screening strategies across all sectors of the older adult population. For example, even where poor mobility may exist, such a restriction may confer access to NHS-funded domiciliary eye examinations.

Despite the limitation of our use of a non-validated measure of patient testing experience, most participants appeared to report a relatively positive experience with the series of tests. Perceived performance was relatively better with the “letter-chart-based” LogMAR test, when compared with the computer-based VF test, likely due to familiarity. Future implementations of visual screening may benefit from patient education materials, digital literacy training, and caregiver involvement to improve engagement and accuracy.

### Limitations

Limitations in this feasibility evaluation include the relatively small sample size from a single slow-turnover unit and the lack of follow-up data to evaluate clinical outcomes. While all patients with identified visual deficits were formally advised to seek primary care eye examinations post-discharge, we have not been able to capture subsequent outcomes within the group. As a result, the benefits of screening require further formal evaluation outside of the scope of our evaluation. The subjective nature of perimetry and its cognitive demands further complicate interpretation, especially in frail inpatient populations. Although efforts were made to control testing conditions, such as reducing ambient lighting, complete control over background luminance was not always achievable.

### Implications

This evaluation has demonstrated how a relatively simple suite of bedside visual screening tools can be feasibly implemented in the inpatient orthogeriatric setting to identify likely visual loss that appears to necessitate ophthalmic assessments and potentially subsequent interventions. The findings highlight several areas for both immediate improvement and long-term service development. First, this evaluation suggests that brief, individualized competency-based training is likely to be adequate to enable non-ophthalmic clinicians to carry out bedside visual screening effectively within the orthogeriatric rehabilitation setting. Currently, there is no integrated visual screening program for older inpatients over the age of 65 in Greater Manchester, despite a high burden of falls-related admissions. The model trialed in this evaluation offers a relatively low-cost and practical framework to help to address this gap. These results, if validated following further formal evaluation of outcomes, could inform local service planning in collaboration with community optometry and support the development of targeted quality improvement initiatives.

To translate these findings into practice, developing an integrated referral pathway, including secondary care clinicians and primary care optometry services, may help overcome the barrier to routine ophthalmic care and improve uptake of GOS. This evaluation showed there is likely to be a high number of the orthogeriatric cohort with relatively poor engagement with primary eye care, despite the majority coming from the least deprived areas of Greater Manchester. Collaborations between hospital teams, community optometry, and NHS commissioners could facilitate periodic visits to rehabilitation units or intermediate care facilities, enabling NHS-subsidized GOS eye examinations for housebound or mobility-impaired patients, reducing the burden on carers to arrange transport and appointments post-discharge.

Improved training for inpatient teams in recognizing key ocular signs may help to support effective triaging, thereby avoiding creating an unnecessary burden on secondary care ophthalmology. Those with likely refractive issues should be directed to community optometry, while older adults with equivocal findings on LCVA or VF testing may also benefit from further assessment from an optometrist first in primary care. Occasionally, prompt and direct referral to ophthalmology may be necessary to avoid unnecessary delays. However, agreements on referral pathways can be supported through interdepartmental teaching, helping to prevent inappropriate referrals by ensuring that patients who are more suitably managed in community optometry are directed there first.

Although this evaluation was conducted within a single orthogeriatric rehabilitation unit, Greater Manchester is one of the largest and most socioeconomically diverse urban populations in the UK, with a population of approximately three million residents. As highlighted in national policy analyses, the region is frequently used as a testbed for population health initiatives due to its demographic diversity and scale, making findings derived from this setting arguably very relevant to other UK conurbations (The King’s Fund, 2024). The challenges identified in this cohort, including limited engagement with community optometry services despite eligibility for NHS-funded sight testing, are therefore likely to be applicable to other regions operating within similar NHS structures. In England, NHS primary eye care is delivered through a well-established community optometry network, which provides free sight testing for eligible groups and enhanced services such as Community Urgent Eyecare Services (CUES). These services are now available across most of England and are specifically designed to support urgent and intermediate eye care within the community, reducing pressure on hospital eye services and improving access to timely assessment (Primary Eyecare, 2024). The findings of this evaluation may therefore inform broader discussions around the integration of structured visual screening into routine orthogeriatric care pathways, supported by existing NHS primary eye care infrastructure. Beyond the UK, this work may serve as a pragmatic model for healthcare systems with established primary eye care services and defined referral pathways to specialist ophthalmology. The underlying principles of the screening approach, including the use of simple, validated tools, brief competency-based training for non-specialist clinicians, and integration within routine inpatient care, are potentially transferable to other high-income healthcare settings. However, the generalizability of this model is likely to vary internationally depending on the availability of primary eye care providers, funding structures, and access to specialist ophthalmology services. International comparisons of eye care delivery emphasize substantial variation in the organization and resourcing of primary eye care, highlighting the need for local adaptation and further evaluation across diverse healthcare systems (OECD, 2025; World Health Organization, 2019).

## Conclusions

This feasibility evaluation demonstrates that structured bedside visual screening can be practicably implemented within an orthogeriatric rehabilitation setting and may provide clinically valuable information when undertaken by trained non-ophthalmic clinicians. The identification of previously unrecognized and potentially modifiable visual deficits in this falls-prone population highlights an important opportunity to strengthen falls prevention strategies within inpatient care. The screening process was generally well received by participants, supporting its acceptability in this setting. These findings support the need for further prospective evaluation to assess downstream clinical outcomes and inform the development of a standardized visual assessment pathway integrated into routine orthogeriatric care.

## Supplementary Material

igag022_Supplementary_Data

## Data Availability

Data are available from the corresponding author upon reasonable request.
