# Supplementary material for: Visual screening in an orthogeriatric rehabilitation setting: a feasibility evaluation
Source: Innov Aging. 2026 Mar 16;10(5):igag022. doi: 10.1093/geroni/igag022 (PMC13082475; doi:10.1093/geroni/igag022)

***Innovation in Aging* Supplementary Material: Yan, Farid, Chillala, & Harper.^.^ Visual screening in an orthogeriatric rehabilitation setting: A Feasibility Evaluation.**

**Data Collection Proforma**

**
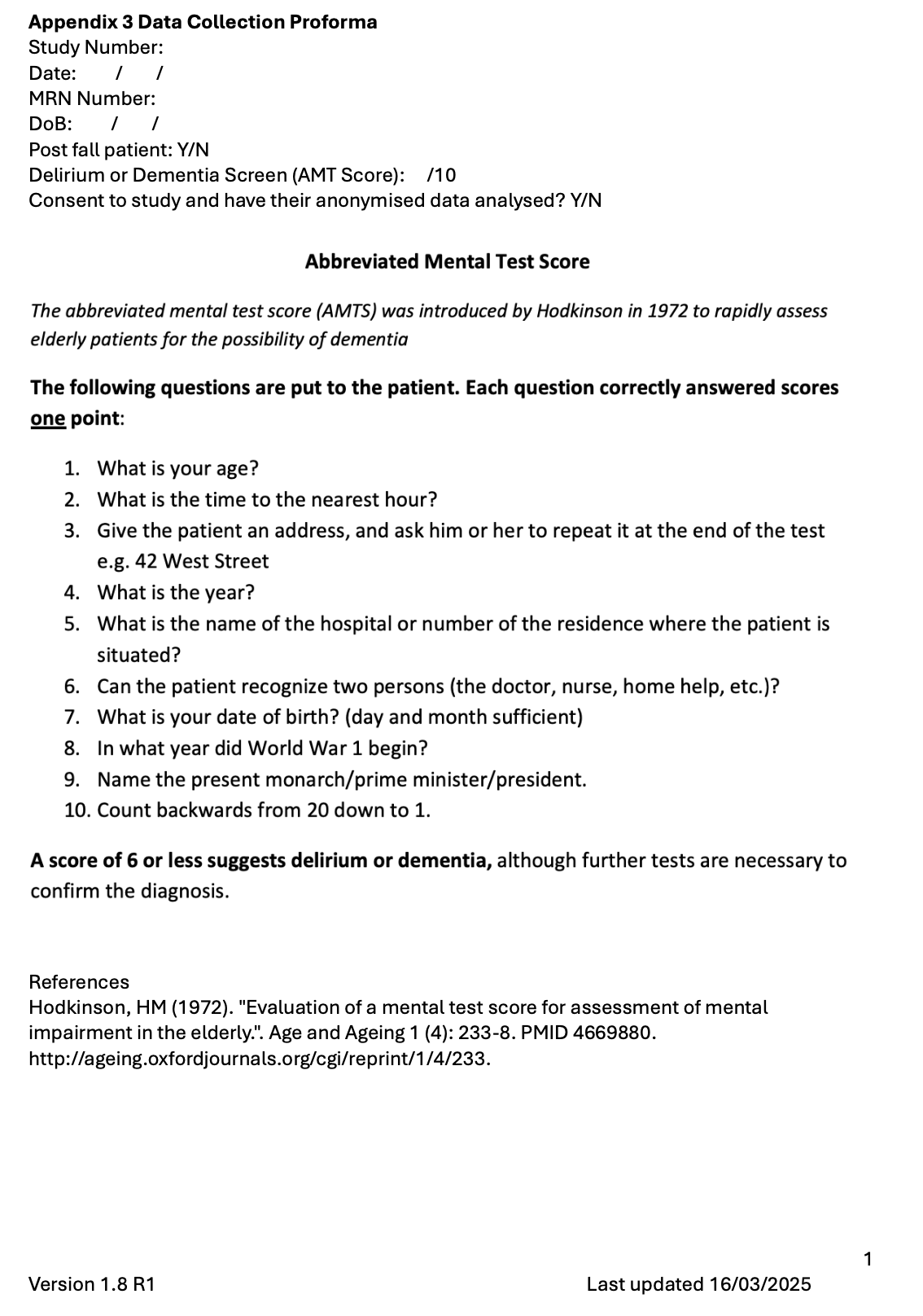
**


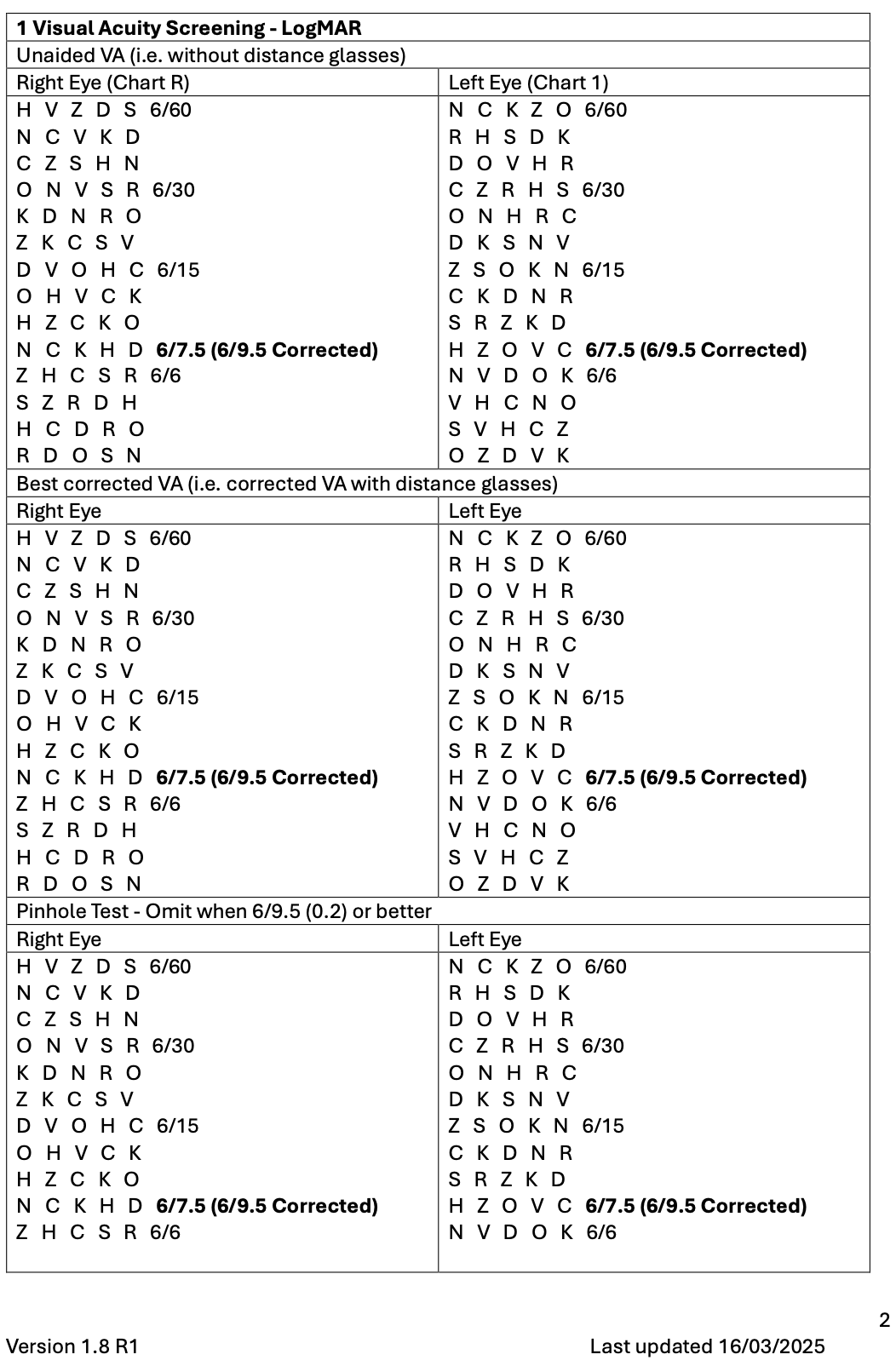


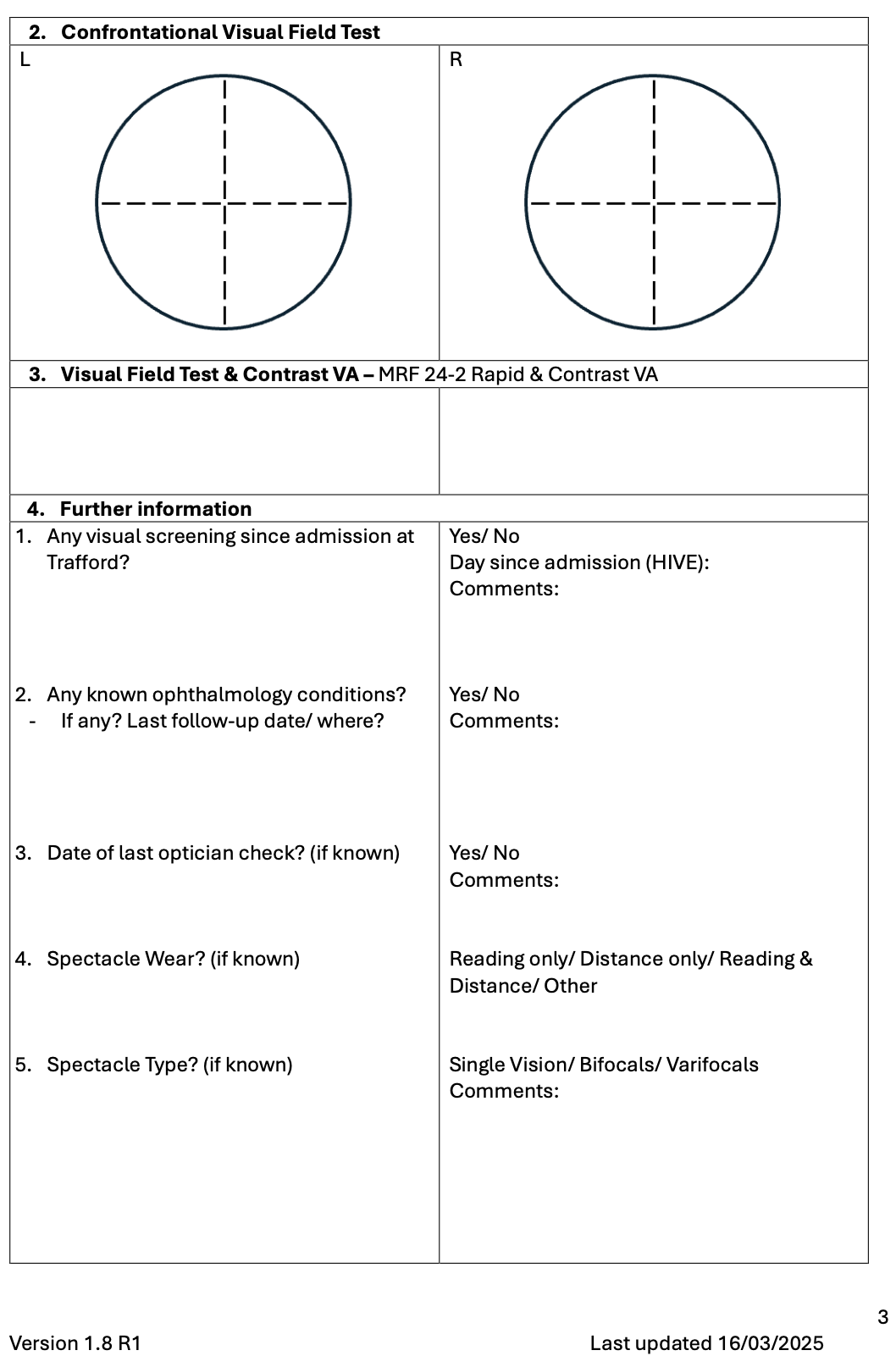


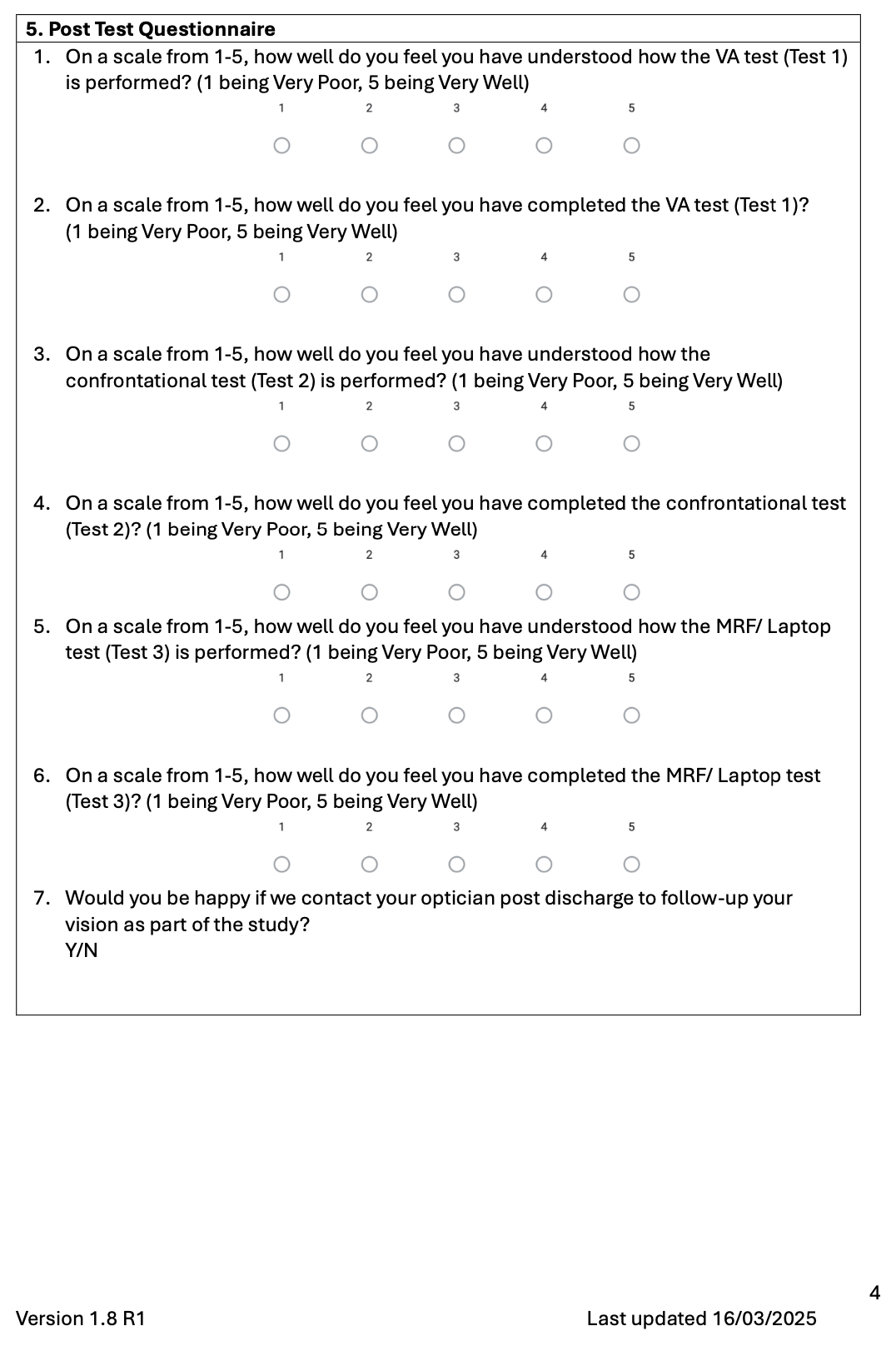

Supplement: igag022_Supplementary_Data [file igag022_supplementary_data.zip › innage suppl Yan, Farid, Chillala, & Harper.docx]
